# Changes in ORF4 of HCoV-229E under different culture conditions

**DOI:** 10.1099/jgv.0.002131

**Published:** 2025-07-10

**Authors:** Yuki Kitai, Shohei Kojima, Aikeda Aishajiang, Miyuki Kawase, Oshi Watanabe, Haruka Yabukami, Rina Hashimoto, Yukiko Akahori, Hiroshi Katoh, Kazuo Takayama, Hidekazu Nishimura, Kazuya Shirato, Makoto Takeda

**Affiliations:** 1 Department of Microbiology, Graduate School of Medicine and Faculty of Medicine, The University of Tokyo, Tokyo, Japan; 2 Virus Research Center, Clinical Research Division, Sendai Medical Center, Sendai, Miyagi, Japan; 3 Laboratory for Developmental Genetics, RIKEN Center for Integrative Medical Sciences, Yokohama, Japan; 4 Department of Virology III, National Institute of Infectious Disease, Tokyo, Japan; 5 Department of Synthetic Human Body System, Medical Research Institute, Institute of Integrated Research, Institute of Science Tokyo, Tokyo, Japan; 6 Center for iPS Cell Research and Application (CiRA), Kyoto University, Kyoto, Japan

**Keywords:** air–liquid interface, HCoV-229E, ORF4

## Abstract

The genome of human coronavirus 229E (HCoV-229E), a causative agent of human respiratory infections, encodes a unique accessory gene, ORF4. Analysis of laboratory strains and clinical specimens has suggested that HCoV-229E acquires truncating mutations in ORF4 under standard laboratory culture conditions. This study confirmed that HCoV-229E from patients with acute respiratory infections harboured a full-length ORF4 (219 amino acids). In contrast, virus stocks derived from the same patients and passaged in conventional cultured cells [LLC-MK2, human embryonic fibroblast (HEF); HEF, HeLa] exhibited truncated ORF4 of various lengths, such as 168, 143 and 16 amino acids. However, when these virus stocks were propagated in human bronchial/tracheal epithelial cells (HBTECs) cultured at the air–liquid interface (ALI), the full-length ORF4 was selected and stably maintained throughout a prolonged observation period. These findings highlight the importance of ORF4 in patients and under physiologically relevant conditions, and the HBTEC-ALI culture system is valuable for analyzing the native properties of HCoV-229E with an intact full-length ORF4.

## Data Availability

The ORF4 sequences generated and analysed in this study have been deposited in GenBank (SMC-H/1121/04, AB691764; SMC-H/1948/04, AB691766; SMC-H/826/04, AB691765).

## Introduction

Human coronavirus 229E (HCoV-229E) is an enveloped virus with a single-stranded RNA genome of ~30 kb and belongs to the genus *Alphacoronavirus* within the family *Coronaviridae*. It is a causative agent of the seasonal common cold [1,2]. Propagation of seasonal coronaviruses (HCoV-229E, -OC43, -NL63 and -HKU1) in cell culture is often difficult, and their biological characteristics remain incompletely understood. The laboratory strain VR-740, widely used in HCoV-229E research, was originally isolated from a patient with respiratory symptoms in 1962 and has since been passaged in various cultured cell lines [2,4]. Full-length genome analysis of the VR-740 strain revealed that, in addition to the conserved coronavirus ORFs (ORF1ab, S, E, M and N), the HCoV-229E genome encodes two additional ORFs, ORF4a and ORF4b, in a partially overlapping configuration that occupies nearly the entire region between the S and E genes [5,6]. In contrast, sequence analysis of clinical specimens revealed that a single, uninterrupted ORF4 is encoded between the S and E genes [7,8]. These findings suggest that the ORF4 region of the VR-740 strain was modified during serial passages in cultured cells. Because conditions in cell lines differ substantially from those of tissues, viruses often acquire adaptive mutations in their genomes during propagation in cell lines. Therefore, careful consideration of cell type and culture conditions is critical for studying the intrinsic properties of viruses replicating in patients. Human bronchial/tracheal epithelial cells (HBTECs) cultured under air–liquid interface (ALI) conditions can provide a more suitable platform than conventional cell lines for virus propagation, by partly mimicking the environment of the human respiratory epithelium. HBTECs have therefore been used to culture various respiratory viruses, including seasonal coronaviruses, influenza viruses, parainfluenza viruses and respiratory syncytial viruses [9,12]. In this study, we systematically examined ORF4 sequences of viruses in patients and their changes during viral propagation in cultured cells, including HBTECs.

## Methods

### Cell culture

LLC-MK2 and human embryonic fibroblast (HEF) cells were cultured in Minimum Essential Medium (MEM; Thermo Fisher Scientific, Waltham, MA, USA) containing 10% FBS (Thermo Fisher Scientific). For virus culture, LLC-MK2 cells were maintained in MEM containing 10 µg ml^−1^ trypsin (Thermo Fisher Scientific), and HEF cells were maintained in MEM supplemented with 2% FBS. All culture mediums contained 100 units per millilitre of penicillin G (Meiji Co., Tokyo, Japan) and 100 µg ml^−1^ of streptomycin (Meiji Co.).

### ALI culture

An ALI culture of HBTECs was prepared as described previously [13,14]. Briefly, HBTEC cells (FC-0035, LIFELINE Cell Technology, Frederick, MD, USA) were plated on 6.5 mm transwell (3470, Corning, One Riverfront Plaza, NY, USA). The next day, the upper medium was removed, and the basal medium was replaced with differentiation medium. Human airway epithelium cultures were generated by culturing cells in an ALI for 4 weeks with a weekly medium change, resulting in well-differentiated, polarized cultures.

### Human iPS cells

The induced pluripotent stem (iPS) cells (1383D6) were provided by Dr. Masato Nakagawa, Kyoto University, and maintained on 0.5 µg cm^−2^ recombinant human laminin 511 E8 fragments (iMatrix-511, Cat# 892 012, Nippi) with StemFit AK02N medium (Cat# RCAK02N, Ajinomoto Healthy Supply). The cells were passed every 6 days. For cell passaging, cell colonies were treated with TrypLE Select Enzyme (Cat# 12563029, Thermo Fisher Scientific) for 10 min at 37 °C and seeded with StemFit AK02N medium containing 10 µM Y-27632 (Cat# 034–24024, FUJIFILM Wako Pure Chemical).

### Respiratory organoids differentiated from human iPS cells

To start the differentiation, human iPS cell colonies were treated with TrypLE Select Enzyme (Cat# 12563029, Thermo Fisher Scientific) for 10 min at 37 °C. After centrifugation, cells were seeded onto Matrigel Growth Factor Reduced Basement Membrane (Cat# 354230, Corning)-coated cell culture plates (2.0×10^5^ cells/4 cm^2^) and cultured for 2 days. The differentiation of the respiratory organoids was performed in serum-free differentiation (SFD) medium, composed of DMEM/F12 (3 : 1) (Cat# 044–29765, FUJIFILM Wako Pure Chemical, and Cat# 11320033, Thermo Fisher Scientific) supplemented with N2 (Cat# 141–08941, FUJIFILM Wako Pure Chemical), B-27 Supplement Minus Vitamin A (Cat# 12587001, Thermo Fisher Scientific), ascorbic acid (50 µg ml^−1^, Cat# ST-72132, STEMCELL Technologies), 1× GlutaMAX (Cat# 35050–061, Thermo Fisher Scientific), 1% monothioglycerol (Cat# 195–15791, FUJIFILM Wako Pure Chemical), 0.05% BSA (Cat# 820024, Sigma-Aldrich) and antibiotics (penicillin and streptomycin). During days 0–1 of differentiation, cells were cultured with SFD medium supplemented with 10 µM Y-27632 (Cat# 034–24024, FUJIFILM Wako Pure Chemical) and 100 ng ml^−1^ recombinant Activin A (Cat# 338-AC-01M, R and D Systems). During days 1–3 of differentiation, cells were cultured with SFD medium supplemented with 10 µM Y-27632 (Cat# 034–24024, FUJIFILM Wako Pure Chemical), 100 ng ml^−1^ recombinant Activin A (Cat# 338-AC-01M, R and D Systems) and 1% FBS. Between days 3 and 5 of differentiation, cells were cultured in SFD medium supplemented with 1.5 µM Dorsomorphin dihydrochloride (Cat# 047–33763, FUJIFILM Wako Pure Chemical) and 10 µM SB431542 (Cat# 037–24293, FUJIFILM Wako Pure Chemical) for 24 h, and then in SFD medium supplemented with 10 µM SB431542 and 1 µM IWP2 (Cat# 04–0034, Stemolecule) for another 24 h. During days 5–12 of differentiation, cells were cultured with SFD medium supplemented with 3 µM CHIR99021 (Cat# 034–23103, FUJIFILM Wako Pure Chemical), 10 ng ml^−1^ human FGF10 (Cat# AF-100–26, PeproTech), 10 ng ml^−1^ human FGF7 (Cat# AF-100–19, PeproTech), 10 ng ml^−1^ human BMP4 (Cat# 120–05ET, PeproTech), 20 ng ml^−1^ human EGF (Cat# AF-100–15, PeproTech) and all-trans retinoic acid (Cat# R2625 ATRA, Sigma-Aldrich). On day 12 of differentiation, cells were dissociated and embedded in the Matrigel Growth Factor Reduced Basement Membrane to generate organoids. During days 12–20 of the differentiation, organoids were cultured in SFD medium containing 3 µM CHIR99021, 10 ng ml^−1^ human FGF10, 10 ng ml^−1^ human FGF7, 10 ng ml^−1^ human BMP4 and 50 nM ATRA. On day 20 of differentiation, organoids were recovered from the Matrigel, and the resulting suspension of organoids (small free-floating clumps) was seeded onto Matrigel-coated cell culture plates. During days 20–30 of differentiation, organoids were cultured in SFD medium containing 50 nM dexamethasone (Cat# S1322, Selleck Chemicals), 0.1 mM 8-bromo-cAMP (Cat# 1140/50, Tocris) and 0.1 mM IBMX (3-isobutyl-1-methylxanthine) (Cat# 095–03413, FUJIFILM Wako Pure Chemical).

### Viruses

Nasal swabs were collected from patients with mild upper respiratory symptoms who presented to clinics and hospitals in Sendai, Japan, in 2004. 229E SMC-H/1121/04 and SMC-H/1948/04 were isolated by the LLC-MK2 cell line. SMC-H/826/04 was isolated by the HEF cell line. The virus isolates were passaged approximately ten times in the respective cell lines used for isolation, followed by two passages in HeLa cells. The sequences of the passaged viruses have already been deposited in GenBank (SMC-H/1121/04, AB691764; SMC-H/1948/04, AB691766; SMC-H/826/04, AB691765) [15].

### Sanger sequencing analysis

Viral RNA was extracted from clinical specimens using QIAamp Viral RNA Mini Kit (Qiagen, Hilden, Germany) according to the manufacturer’s instructions. The extracted RNA was reverse-transcribed and amplified using the PrimeScript^™^ II High Fidelity RT-PCR (reverse transcription PCR) Kit (Takara Bio Inc., Shiga, Japan). The PCR products were purified using NucleoSpin^®^ Gel and PCR Clean-up (Takara Bio Inc.) and then subjected to Sanger sequencing analysis (Eurofins Genomics, Tokyo, Japan). Primer sets (5′ TGCTGCGGCTTCTTTAGTTG 3′, 5′ ATAAGCACCACACACCAGAG 3′) were used for PCR and sequencing. Sequence data were analysed using mega 11 (version 11.0.13) and Benchling (Benchling, San Francisco, CA, USA).

### Next-generation sequencing analysis

For next-generation sequencing (NGS) of the virus stocks, libraries were prepared using the NEBNext Ultra II RNA Library Prep Kit for Illumina (New England Biolabs, Ipswich, MA, USA), following the manufacturer’s instructions. Indexed libraries were sequenced for 2×150 cycles on a DNBSEQ-G400 sequencer at AZENTA/GENEWIZ (Chelmsford, MA, USA). Reads were trimmed and then *de novo* assembled and/or mapped to the respective reference sequences using the CLC Genomics Workbench (v21.0.4, Qiagen) with default settings.

Nucleic acids were extracted from apical washes using QIAamp 96 Virus QIAcube HT Kit (Qiagen) following the manufacturer’s instructions, except that the elution step was performed by centrifugation. RNA samples were reverse-transcribed into cDNA using the Verso cDNA Synthesis Kit and a primer, 229E_RT_primer, which specifically binds to the envelope gene of 229E strains. ORF4 was amplified from the cDNA by 15 cycles of PCR using primers ORF4_f and ORF4_r. These primers were designed to bind proximal to the two mutations in ORF4 (i.e. the C-to-T substitution and the deletion of TA) so that both mutation sites could be sequenced within the 75 bp×2 paired-end reads. SP1 and SP2 tags for Illumina sequencing were added to the amplicons by five cycles of PCR with primers SP1_primer and SP2_primer. Subsequently, the adapter sequences for Illumina sequencing were added to the amplicons by ten cycles of PCR with primers carrying adapter sequences and finally generated the NGS libraries. All PCRs were performed using PrimeSTAR GxL DNA Polymerase. The libraries were sequenced on an Illumina MiSeq platform using the MiSeq Reagent Kit v3 (75 bp×2 paired-end reads). See Table S5 (available in the online Supplementary Material) for primer sequences. Fifteen bases were trimmed from the 5′- and 3′-ends of the NGS reads. After trimming, reads containing three or more ‘N’ bases, as well as reads carrying at least one base having a quality score below 20, were filtered out. Then, the occurrences of the two mutations, the C-to-T substitution and the deletion of TA, in the NGS reads were counted using a custom Python script, ORF4_mutation_detection.py, deposited on GitHub (https://github.com/shohei-kojima/2025_ORF4_mutations).

## Results

**Fig. 1.**
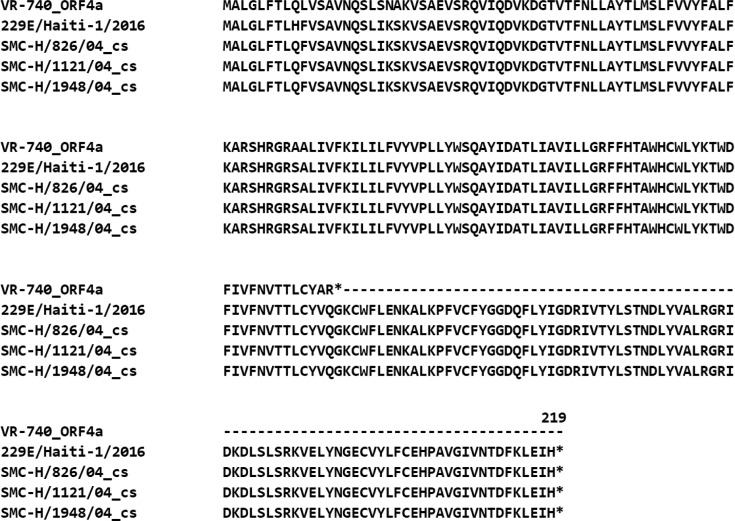
Alignment of ORF4 sequences in clinical specimens. The ORF4 sequence in clinical specimens was determined using Sanger sequencing and aligned with Clustal W. For comparison, the ORF4a of the laboratory strain (VR-740) and the ORF4 of the clinical strain (229E/Haiti-1/2016) were also aligned. The GenBank accession numbers for each sequence are as follows: VR-740 (OK625404), 229E/Haiti-1/2016 (MF542265), SMC-H/1121/04 (AB691764), SMC-H/1948/04 (AB691766) and SMC-H/826/04 (AB691765).

Virus Research Center, Sendai Medical Center (SMC) (Sendai, Miyagi, Japan), isolates and propagates multiple respiratory viruses from patients with acute respiratory infections using microplate-based virus isolation systems [16]. In 2004, using these systems, three HCoV-229E strains were obtained from three different patients with mild upper respiratory symptoms. Two strains (Sendai-H/1121/04 and Sendai-H/1948/04) were isolated and propagated using LLC-MK2 cells, and one strain (Sendai-H/826/04) using HEF cells. To avoid confusion with Sendai virus, they were termed SMC-H/1121/04, SMC-H/1948/04 and SMC-H/826/04, respectively, in this study. To determine the original ORF4 sequences of viruses replicating in patients, viral RNA was extracted from the patients’ throat swabs, and the ORF4 gene was amplified by PCR and sequenced by Sanger sequencing. The results showed that all three strains possessed an ORF4 gene 660 bases in length, occupying almost the entire region between the S and E genes. This 660-base region encodes a single ORF4 protein consisting of 219 amino acids, similar to that of 229E/Haiti-1/2016 reported previously (MF542265) (Fig. 1) [17].

**Fig. 2.**
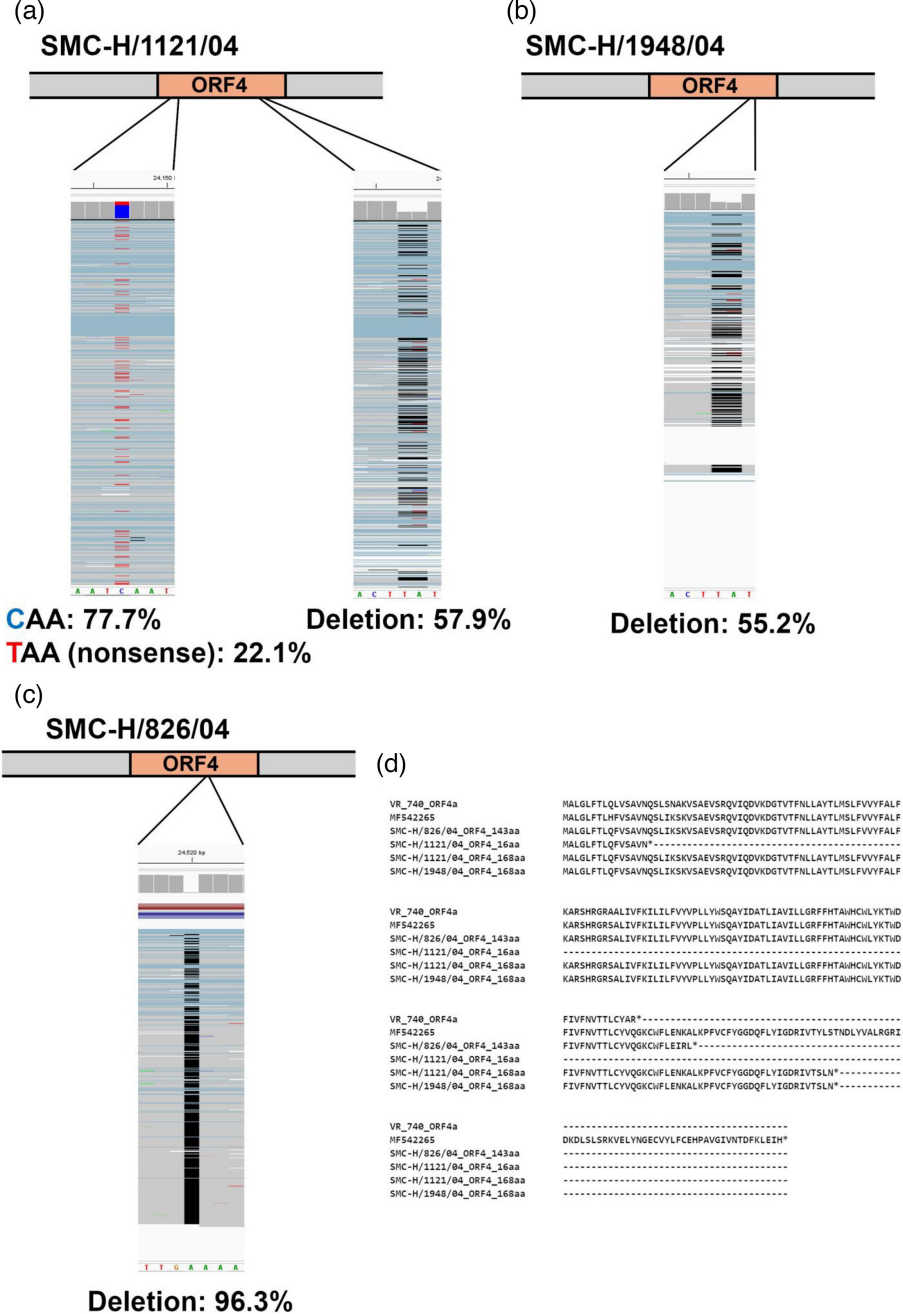
Analysis of ORF4 sequences in cell-adapted HCoV-229E. The ORF4 gene of HCoV-229E isolates passed in LLC-MK2 or HEF cells followed by HeLa cells was analysed using NGS. The sequencing reads were mapped to the ORF4 gene of 229E/Haiti-1/2016 (MF542265), which was used as the reference. (a–c) Mutation sites in SMC-H/1121/04, SMC-H/1948/04 and SMC-H/826/04 were visualized using Integrative Genomics Viewer, respectively. A mixture of red and blue indicates a mix of C and T bases. Black indicates a base deletion. (**d**) The ORF4 sequences of cell-adapted HCoV-229E strains were aligned using Clustal W. For comparison, the ORF4a of the laboratory strain VR-740 and the ORF4 of the clinical strain 229E/Haiti-1/2016 were also included in the alignment.

Virus stocks of the three strains were obtained through approximately ten passages in LLC-MK2 or HEF cells, followed by two additional passages in HeLa cells. NGS analysis revealed that a two-base deletion occurred in the ORF4 region in 57.9% of the viral population of the SMC-H/1121/04 strain (Fig. 2a). This deletion caused a frameshift, truncating ORF4 from 219 amino acids to 168 amino acids (SMC-H/1121/04_ORF4_168aa) (Fig. 2d). Furthermore, 22.1% of the population harboured a nonsense mutation in ORF4, resulting in a severely truncated ORF4 of only 16 amino acids (SMC-H/1121/04_ORF4_16aa) (Fig. 2a, d). In the SMC-H/1948/04 strain, 55.2% of the population (SMC-H/1948_ORF4_168aa) exhibited the same two-base deletion as observed in the SMC-H/1121/04 strain, causing a frameshift and truncation of ORF4 to 168 amino acids (Fig. 2b, d). In the SMC-H/826/04 strain, 96.3% of the population had a single-nucleotide deletion in the ORF4 gene, resulting in a frameshift that truncated ORF4 to 143 amino acids (SMC-H/826/04_ORF4_143aa) (Fig. 2c, d). These results indicate that propagation in cultured cells leads to truncating mutations in ORF4. Notably, the SMC-H/1121/04 and SMC-H/1948/04 stocks, which had undergone extensive passages in LLC-MK2 cells, exhibited increased proportions of truncated ORF4 (data not shown).

**Fig. 3.**
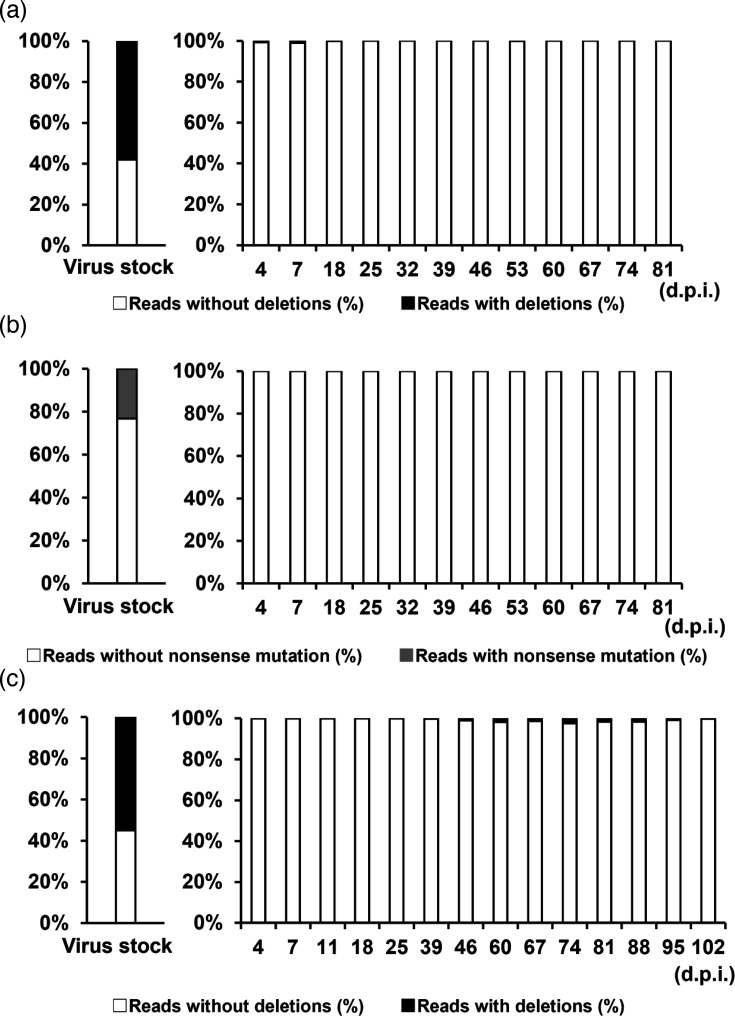
Effect of ALI culture on ORF4 gene mutations in HCoV-229E. SMC-H/1121/04 and SMC-H/1948/04, previously passaged in LLC-MK2 and HeLa cells, were used to infect HBTECs cultured under ALI conditions. The ORF4 genes of viruses collected at each time point were analysed using NGS. (**a, b**) Proportions of two-base deletions and nonsense mutations in SMC-H/1121/04. (**c**) Proportion of two-base deletions in SMC-H/1948/04. White bars indicate mutation-free reads, black bars indicate reads with two-base deletions and grey bars indicate reads with nonsense mutations.

The SMC-H/1121/04 and SMC-H/1948/04 virus stocks with ORF4 truncations were propagated in HBTEC-ALI cultures. Apical washes from HBTEC-ALI cultures infected with these virus stocks were collected every 7–10 days, and viral RNAs were extracted. Real-time RT-PCR confirmed significant levels of viral RNA in the apical washes for up to ~100 days post-infection, indicating sustained HCoV-229E infection in HBTEC-ALI cultures (Fig. S1). The ORF4 gene was amplified by RT-PCR, and mutation frequencies in the ORF4 gene were analysed by NGS. Although ORF4 mutations (nonsense mutation or base deletions) were detected in ~20–60% of the virus stock prior to ALI culture, in both SMC-H/1121/04 and SMC-H/1948/04, the full-length ORF4 sequence was almost exclusively detected as early as 4 days post-infection and remained dominant thereafter (Fig. 3a–c and Tables S1–S3). In SMC-H/1948/04, the detection of mutation reads slightly increased in samples from days 46 to 95 but eventually became almost undetectable.

The infectivity of apical wash samples was evaluated by TCID_50_ assays using LLC-MK2 cells (Table S4). An apparent cytopathic effect (CPE) was observed in the SMC-H/1121/04-infected cells, while no CPE was detected in the SMC-H/1948/04-infected cells. Subsequent sequence analysis showed that the SMC-H/1121/04 had reacquired the same nonsense mutation in ORF4, leading to truncation, whereas the SMC-H/1948/04 retained the full-length ORF4. Additionally, infection experiments using iPS cell-derived respiratory organoids demonstrated that the nonsense mutation and two-base deletion in ORF4 observed in the virus stock were resolved, and the full-length ORF4 sequence became predominant in the viral population, further supporting the findings obtained in HBTEC-ALI cultures (Fig. S2). These results suggest that strains containing the full-length ORF4 of HCoV-229E selectively proliferate in HBTEC-ALI culture and that a truncation of ORF4 is required for HCoV-229E to replicate with apparent CPE in LLC-MK2 cells.

## Discussion

Analysis of the ORF4 gene in the VR-740 strain and clinical specimens has suggested that HCoV-229E frequently acquires truncating mutations in the ORF4 gene [5,7, 8]. In this study, we compared the ORF4 sequences of viruses propagated in conventional cell lines and HEF and found that truncating mutations commonly occur in these cell types. A key observation was that viruses with an intact ORF4 sequence were almost exclusively propagated in the HBTEC-ALI culture system. These findings suggest that full-length ORF4 is dispensable – and possibly disadvantageous – for replication in conventional cell cultures, but critically important for viral growth in HBTEC-ALI cultures, which partially mimic the physiological environment of the human respiratory epithelium.

The length of truncated ORF4 varied among virus strains: SMC-H/1121/04 (16 or 168 amino acids), SMC-H/1948/04 (168 amino acids) and SMC-H/826/04 (143 amino acids) (Fig. 2d). Dijkman *et al*. reported that the ORF4 length in the laboratory strains VR-740 and HC-LC was also shortened to 133 and 86 amino acids, respectively [7]. Zhang *et al*. reported that the 133-amino acid ORF4 (ORF4a) encoded by the VR-740 strain functions as a viroporin in cultured cells; however, further investigation is needed to determine whether the full-length ORF4 has the same function or possesses distinct properties [18]. The ORF3 of porcine epidemic diarrhoea virus (PEDV), a homologue of HCoV-229E ORF4, has exhibited anti-IFN activity, suggesting that ORF4 may possess a similar function [19]. Furthermore, since the strength of the IFN response to viral infection differs between immortalized and primary cells, this response may serve as a selective pressure, favouring the retention of ORF4 in cells with strong IFN responses and its deletion in cells with weak IFN responses [20]. HCoV-NL63, PEDV and transmissible gastroenteritis virus, which belong to the same genus, *Alphacoronavirus*, as HCoV-229E, undergo mutations in ORF3, a homologue of ORF4, after long-term passage in cultured cells [21,22]. HCoV-NL63 lacking ORF3 exhibits reduced replication efficiency in primary human airway epithelial cells under ALI conditions [23]. These findings suggest that ORF3 and ORF4 in *Alphacoronavirus* contribute to efficient viral replication *in vivo*. All clinical specimens analysed in this study were derived from patients with mild respiratory illness, and no apparent correlation between ORF4 status and disease severity was observed. Further studies including specimens from patients with more diverse clinical outcomes would be required to clarify the potential clinical significance of ORF4.

The isolation and propagation of viruses using cultured cells are fundamental to conduct virus research. However, viruses, which have been adapted to grow in cultured cells, often exhibit different phenotypes from the original ones in patients or infected host animals. For HCoV-229E, truncating mutations in ORF4 represent a specific example. Similarly, when HCoV-229E adapts to cultured cells, mutations occur in the spike protein, altering its preferred cell entry mechanism from transmembrane serine protease 2-dependent to cathepsin-dependent entry [24]. Our results suggest that HBTEC-ALI serves as a valuable platform for studying HCoV-229E. In addition to HBTEC-ALI cultures, we also observed preferential retention of full-length ORF4 in human respiratory organoids derived from iPS cells. These data provide additional evidence showing that full-length ORF4 is favored in physiologically relevant epithelial models. In conclusion, research utilizing the HBTEC-ALI culture system can provide new insights into *in vivo* propagation of HCoV-229E harbouring the intact full-length ORF4.

## Supplementary material

10.1099/jgv.0.002131 Uncited Supplementary Material 1.

## Abbreviations

ALI: air–liquid interface
CPE: cytopathic effect
HBTEC(s): human bronchial/tracheal epithelial cell(s)
HCoV-229E: human coronavirus 229E
HEF: human embryonic fibroblast
iPS: induced pluripotent stem
MEM: Minimum Essential Medium
NGS: next-generation sequencing
PEDV: porcine epidemic diarrhoea virus
RT-PCR: reverse transcription PCR
SFD: serum-free differentiation
SMC: Sendai Medical Center
