# Supplementary material for: Changes in ORF4 of HCoV-229E under different culture conditions
Source: J Gen Virol. 2025 Jul 10;106(7):002131. doi: 10.1099/jgv.0.002131 (PMC12282251; doi:10.1099/jgv.0.002131)
Supplement: Uncited Supplementary Material 1. [file jgv-106-02131-s001.pdf]

Figure S1

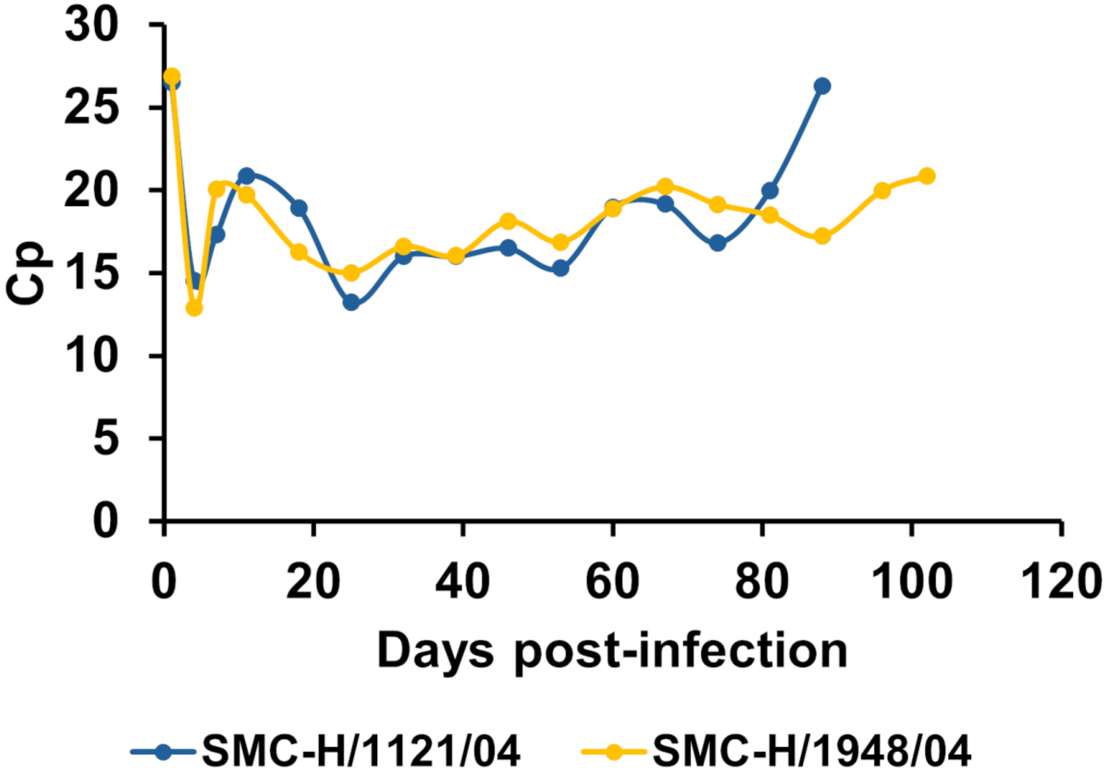

30
25
20
15
10
5
0
Cp
0
20
40
60
80
100
120
Days post-infection
SMC-H/1121/04
SMC-H/1948/04

Figure S2

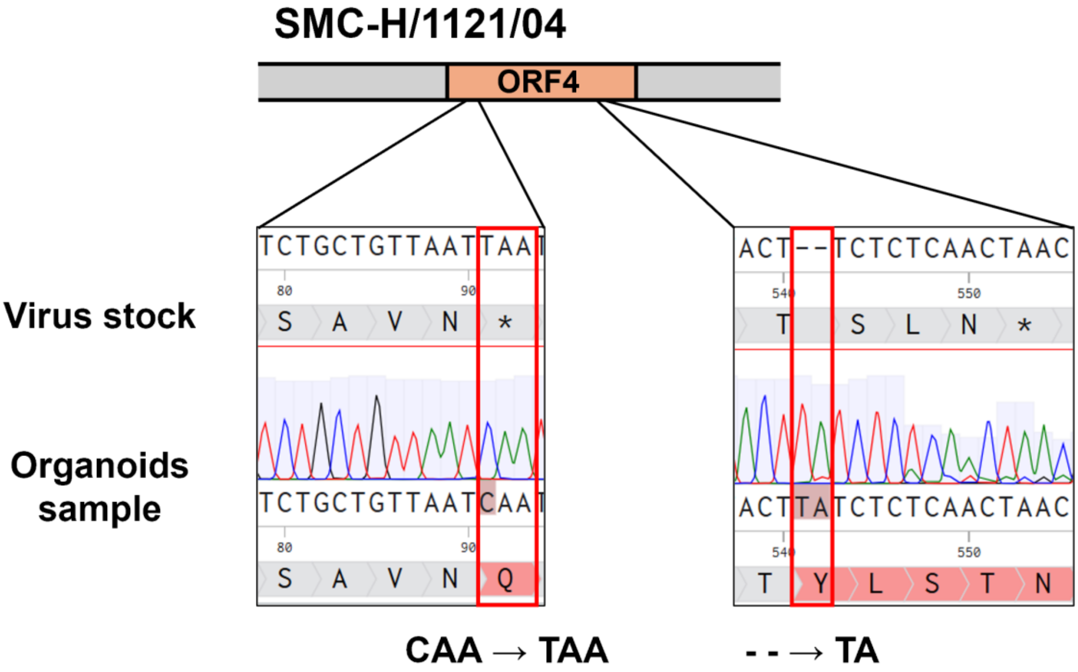
SMC-H/1121/04
ORF4
Virus stock
Organoids sample
TCTGCTGTTAATTAAT
80
90
S A V N *
TCTGCTGTTAATCAAT
S A V N Q
ACT--TCTCTCAACTAAC
540
550
T S L N *
ACTTATCTCTCAACTAAC
T Y L S T N
CAA → TAA
- - → TA

Table S1 NGS data of 229E/1121/04 ORF4 nonsense mutation in ALI culture

Samples	Reads without nonsense mutation	Reads with nonsense mutation	Other reads	Reads without nonsense mutation (%)	Reads with nonsense mutation (%)	Other reads (%)
**1121 4 dpi**	**214,405**	**28**	**625**	**99.70**	**0.01**	**0.29**
**1121 7 dpi**	**295,932**	**43**	**1,118**	**99.61**	**0.01**	**0.38**
**1121 18 dpi**	**472,957**	**83**	**1,277**	**99.71**	**0.02**	**0.27**
**1121 25 dpi**	**380,086**	**62**	**912**	**99.74**	**0.02**	**0.24**
**1121 32 dpi**	**440,109**	**74**	**1,025**	**99.75**	**0.02**	**0.23**
**1121 39 dpi**	**162,409**	**23**	**383**	**98.75**	**0.01**	**0.24**
**1121 46 dpi**	**519,292**	**87**	**1,376**	**99.72**	**0.02**	**0.26**
**1121 53 dpi**	**551,960**	**89**	**1,302**	**99.75**	**0.02**	**0.24**
**1121 60 dpi**	**463,248**	**92**	**1,391**	**98.68**	**0.02**	**0.30**
**1121 67 dpi**	**539,321**	**107**	**2,060**	**99.60**	**0.02**	**0.38**
**1121 74 dpi**	**427,557**	**69**	**1,183**	**99.74**	**0.01**	**0.25**
**1121 81 dpi**	**40,133**	**10**	**474**	**98.81**	**0.02**	**1.16**

Table S2 NGS data of 229E/1121/04 ORF4 two-base deletions in ALI culture

Samples	Reads without deletions	Reads with deletions	Other reads	Reads without deletions (%)	Reads with deletions (%)	Other reads (%)
1121 4 dpi	209,344	1,600	4,114	97.34	0.74	1.91
1121 7 dpi	291,559	3,184	2,350	98.14	1.07	0.79
1121 18 dpi	471,850	381	2,086	99.48	0.08	0.44
1121 25 dpi	379,369	84	1,607	99.56	0.02	0.42
1121 32 dpi	439,254	50	1,904	99.56	0.01	0.43
1121 39 dpi	160,900	40	1,875	98.82	0.02	1.15
1121 46 dpi	518,575	117	2,063	99.58	0.02	0.40
1121 53 dpi	550,718	488	2,145	99.52	0.09	0.39
1121 60 dpi	459,118	92	5,521	98.79	0.02	1.19
1121 67 dpi	524,873	90	16,525	96.93	0.02	3.05
1121 74 dpi	463,025	68	10,716	97.72	0.01	2.26
1121 81 dpi	39,945	2	670	98.35	0.00	1.65

Table S3 NGS data of 229E/1948/04 ORF4 two-base deletions in ALI culture

Samples	Reads without deletions	Reads with deletions	Other reads	Reads without deletions (%)	Reads with deletions (%)	Other reads (%)
1948 4 dpi	234,739	176	1,061	99.48	0.07	0.45
1948 7 dpi	249,036	238	1,728	99.22	0.09	0.69
1948 11 dpi	301,174	111	3,154	98.93	0.04	1.04
1948 18 dpi	233,705	52	1,305	99.42	0.02	0.55
1948 25 dpi	97,186	16	891	99.08	0.02	0.91
1948 39 dpi	281,669	493	3,170	98.72	0.17	1.11
1948 46 dpi	184,032	1,707	1,773	98.14	0.91	0.95
1948 60 dpi	217,282	3,729	12,343	93.11	1.60	5.29
1948 67 dpi	113,617	1,388	4,384	95.17	1.16	3.67
1948 74 dpi	441,605	10,750	5,089	96.54	2.35	1.11
1948 81 dpi	436,690	6,855	9,047	96.49	1.51	2.00
1948 88 dpi	336,231	5,271	9,760	95.72	1.50	2.78
1948 95 dpi	335,078	2,758	48,763	86.67	0.71	12.61
1948 102 dpi	207,307	360	47,365	81.29	0.14	18.57

Table S4 $TCID_{50}$ titer of ALI culture samples

Sample	Viral titer ($TCID_{50}$/ml)
1121<br>4 dpi	$10^{6.8}$
1121<br>81 dpi	$10^{5.6}$
1948<br>4 dpi	N.D.
1948<br>102 dpi	N.D.

N.D., not detected

Table S5 Primer information for NGS analysis

Purpose	Primer name	Primer sequnce
Reverse transcription	229E_RT_primer	AGCACCACACACCAGAGTAG
Amplification of ORF4	ORF4_f	ATGGCTCTAGGTTTGTTCAC
Amplification of ORF4	ORF4_r	CTCTAAGAGCAACGTACAAATCGT
Adding SP tag	SP1_primer	TCGTCGGCAGCGTCAGATGTGTATAAGAGACAGATGGCTCTAGGTTTGTTCAC
Adding SP tag	SP2_primer	GTCTCGTGGGCTCGGAGATGTGTATAAGAGACAGCTCTAAGAGCAACGTACAAATCGT
Adding adapter sequence	Adapter_f1	AATGATACGGCGACCACCGAGATCTACACGTAAGGAGTCGTCGGCAGCGTC
Adding adapter sequence	Adapter_f2	AATGATACGGCGACCACCGAGATCTACACACTGCATATCGTCGGCAGCGTC
Adding adapter sequence	Adapter_f3	AATGATACGGCGACCACCGAGATCTACACAAGGAGTATCGTCGGCAGCGTC
Adding adapter sequence	Adapter_f4	AATGATACGGCGACCACCGAGATCTACACCTAAGCCTTCGTCGGCAGCGTC
Adding adapter sequence	Adapter_f5	AATGATACGGCGACCACCGAGATCTACACCGTCTAATTCGTCGGCAGCGTC
Adding adapter sequence	Adapter_r1	CAAGCAGAAGACGGCATACGAGATTTCTGCCTGTCTCGTGGGCTCGG
Adding adapter sequence	Adapter_r2	CAAGCAGAAGACGGCATACGAGATGCTCAGGAGTCTCGTGGGCTCGG
Adding adapter sequence	Adapter_r3	CAAGCAGAAGACGGCATACGAGATAGGAGTCCGTCTCGTGGGCTCGG
Adding adapter sequence	Adapter_r4	CAAGCAGAAGACGGCATACGAGATCATGCCTAGTCTCGTGGGCTCGG
Adding adapter sequence	Adapter_r5	CAAGCAGAAGACGGCATACGAGATGTAGAGAGGTCTCGTGGGCTCGG
Adding adapter sequence	Adapter_r6	CAAGCAGAAGACGGCATACGAGATCAGCCTCGGTCTCGTGGGCTCGG
